# Keep in touch: a perspective on the mitochondrial social network and its implication in health and disease

**DOI:** 10.1038/s41420-023-01710-9

**Published:** 2023-11-16

**Authors:** Silvia Barabino, Silvia Lombardi, Mara Zilocchi

**Affiliations:** Department of Biotechnology and Biosciences, University of Milano-Bicocca, 20126 Milan, Italy

**Keywords:** Cell biology, Neuroscience

## Abstract

Mitochondria have been the focus of extensive research for decades since their dysfunction is linked to more than 150 distinct human disorders. Despite considerable efforts, researchers have only been able to skim the surface of the mitochondrial social complexity and the impact of inter-organelle and inter-organ communication alterations on human health. While some progress has been made in deciphering connections among mitochondria and other cytoplasmic organelles through direct (i.e., contact sites) or indirect (i.e., inter-organelle trafficking) crosstalk, most of these efforts have been restricted to a limited number of proteins involved in specific physiological pathways or disease states. This research bottleneck is further narrowed by our incomplete understanding of the cellular alteration timeline in a specific pathology, which prevents the distinction between a primary organelle dysfunction and the defects occurring due to the disruption of the organelle’s interconnectivity. In this perspective, we will (i) summarize the current knowledge on the mitochondrial crosstalk within cell(s) or tissue(s) in health and disease, with a particular focus on neurodegenerative disorders, (ii) discuss how different large-scale and targeted approaches could be used to characterize the different levels of mitochondrial social complexity, and (iii) consider how investigating the different expression patterns of mitochondrial proteins in different cell types/tissues could represent an important step forward in depicting the distinctive architecture of inter-organelle communication.

## Introduction

Over the past few decades, mitochondrial homeostasis has been the focus of many studies trying to decipher its role in human health and disease, along with determining the different cellular conditions that lead to a shift in mitochondrial physiological functions. With the ongoing increase in the number of pivotal proteins for mitochondrial homeostasis [1–3], as well as the creation of curated collections of mammalian mitochondrial proteins (such as MitoCarta 3.0 [4], MitoMiner v4.0 [5], and UniProt [6]), it has become clear that we have just begun to scratch the surface of the complexity of these cytoplasmic organelles. In particular, the molecular mechanisms controlling their plasticity, dynamicity, and ability to integrate and respond to cellular, environmental, and developmental stimuli have yet to be unraveled. This knowledge gap is further exacerbated by the numerous mitochondrial proteins that lack a clear characterization (i.e., orphan proteins) [2] due to their selective expression in specific tissues or developmental states, as well as the technical limitation of current proteomics approaches (which are not able to detect low-abundance proteins) [7].

Nevertheless, the exponential increase in the number of published papers on mitochondrial homeostasis has highlighted the extraordinary “social” nature of these cytoplasmic organelles [8–10]. Mitochondria are constantly communicating and cooperating not only with each other but also with other cellular compartments. The disruption of this crosstalk can lead to deep cellular damage and the onset of diseases [8]. Another level of “social” complexity is added when we consider the diversity of mitochondrial shapes and functions in different human tissues and organs [11], as well as their ability to release extracellular signals (e.g., mitokines) that can influence the whole-body physiology [9, 10].

Here, we will summarize the available evidence highlighting the social connections among mitochondria and other organelles/tissues, as well as their impairment in human diseases—with a particular focus on neurodegenerative disorders. Lastly, we will describe the available techniques that could be adapted for the characterization of the mitochondrial social network at different levels of biological complexity.

### Mitochondrial communications across the cell

Communication among different types of cytoplasmic organelles occurs via the exchange of metabolites, proteins, and ions, which is influenced by the cellular status (e.g., cell cycle stage, differentiation, and activation of internal pathways), as well as other external stimuli [12, 13].

**Fig. 1:**
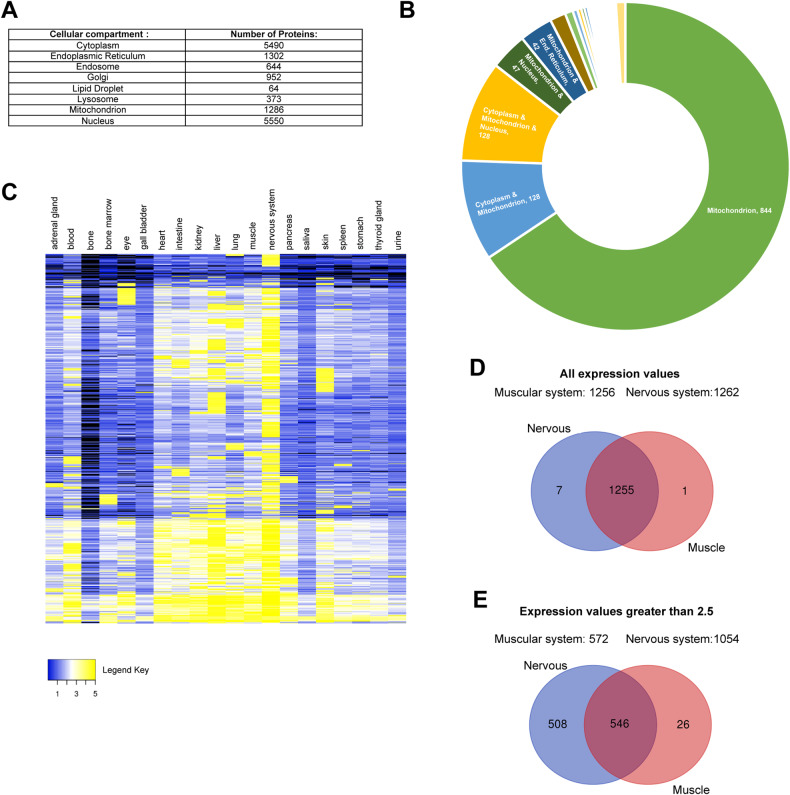
Mitochondrial protein expression in cells and tissues. **A** Number of proteins downloaded from the UniProt Database pertaining to eight different cytoplasmic compartments. **B** Chart representing the number of proteins localizing only in the mitochondrion or also in other cytoplasmic compartments. **C** Heatmap representing the expression of 1275 UniProt mitochondrial proteins according to the TISSUES database. Yellow color is used for highly expressed mitochondrial proteins, while blue for the ones expressed at lower levels. Black is used to highlight the proteins not expressed in that tissue (i.e., no value in the TISSUES database—see Supplementary Table 3). **D** and **E** Venn diagrams representing the total mitochondrial proteins (**D**, all expression values) or the mitochondrial proteins highly expressed (**E**, expression value greater than 2.5) in the muscle vs. nervous system, according to the TISSUES database (see Supplementary Table 3).

Besides the adaptation mechanisms that allow the information exchange among mitochondria (e.g., inter-mitochondrial junctions, mitochondrial nanotunnels, and mitochondrial fusion) [9], these cytoplasmic organelles have extensive intra-cellular social connections [8]. We initially approached this social network by considering the proteins belonging to eight different cellular compartments (Fig. 1A) and verifying which of these polypeptides were common between two or more cytoplasmic organelles (i.e., dual or multiple localization). To do this, we downloaded the protein IDs of each specific cellular compartment using the UniProt database [6] (which was accessed on: March 24th, 2023) (Fig. 1A, Supplementary Table 1, and Supplementary Information) and highlighted the proteins with multiple localization (Supplementary Table 2). By focusing exclusively on the mitochondrial component, we found that only 844 (65.6%) of the total 1286 mitochondrial proteins are uniquely localized in the mitochondrion, while the remaining 442 (34.4%) mitochondrial proteins have dual or multiple cellular localization (Fig. 1B and Supplementary Table 2), thus emphasizing the complexity of the mitochondrial protein interplay at the cellular level.

In this social context, the majority of proteins are shared among (**i**) mitochondrion-nucleus (47 proteins), (**ii**) mitochondrion-cytoplasm (128 proteins), and (**iii**) mitochondrion-cytoplasm-nucleus (128 proteins) (Supplementary Table 2). This suggests that mitochondrial, cytoplasmic, and nuclear fitness strictly depends on the ability of these compartments to communicate with each other [14, 15]. In particular, nuclear gene expression strictly depends on mitochondrial energy production, while the majority of mitochondrial proteins are encoded by the nucleus with only 13 proteins encoded by the mitochondrial DNA (mtDNA) [15]. Therefore, a perfect synchronization of expression, transcription, translation, and import of mitochondrial proteins is necessary to (**i**) maintain a stoichiometric balance between nuclear and mitochondrial genome, (**ii**) adapt to the cellular needs by increasing (i.e., mitochondrial biogenesis) or decreasing (i.e., mitophagy) the mitochondrial volume density, and (**iii**) guarantee the optimal function of both cytoplasmic organelles.

The mitochondrion-lysosome and mitochondrion-endoplasmic reticulum axes are also important for the maintenance of proper cellular homeostasis. Malfunctions of one of these cytoplasmic organelles will backlash on the other component, determining the onset of pathological processes [8]. This is particularly evident in lysosomal storage diseases (LSDs), which are characterized by structural and functional mitochondrial impairments [16], as well as in other neurodegenerative disorders caused by mutations in mitochondrial genes, which are characterized by endoplasmic reticulum perturbations [17, 18].

Despite great progress has been made in deciphering mitochondrial social connections (which are broadly described in other reviews [8, 9, 13, 19]), researchers have just begun to investigate the functional integration of cellular compartments and how this crosstalk differs between cell and tissue types, branching out from the single-cell confinement to extracellular signals.

### Mitochondrial communications beyond the cellular boundary

Since mitochondrial functions are dynamically influenced by their surrounding cellular environment, it is not surprising that the mitochondrial protein composition strictly depends on the type of cell in which they are found [20]. This diversification allows not only to meet the specific energetic and metabolic requirements but also to maintain the functional specificity typical of every organ and tissue. This is particularly evident when we consider the ability of mitochondria to self-organize and synchronize their activity in a cell-type-specific manner [21–24], thus fulfilling the bioenergetic requirements of each specific human organ/tissue [25]. For instance, the remarkable heterogeneity of the mitochondrial proteome, morphology, and functionality found in neurons is necessary to meet the different metabolic demands of these polarized cells [26].

To offer an overview of this specialization, we imported all 1286 mitochondrial proteins found in the UniProt database (Supplementary Table 1) into the protein query function of the STRING Cytoscape app [27]. By using this function, we automatically generated a protein interaction network consisting of 1275 nodes (the network is not shown, but it can be easily reproduced by importing the UniProt mitochondrial proteins into the protein query function of the STRING Cytoscape app). We then visualized their differential expression across 20 human tissues using the STRING Cytoscape app, which allows to retrieve the protein expression evidence from the TISSUES database [28] (Fig. 1C, Supplementary Table 3, and Supplementary Information). This global overview (Fig. 1C) of mitochondrial protein expression highlights the importance of the cellular environment on the mitochondrial proteomics profile specification. To further emphasize this specialization, we decided to focus on the mitochondrial protein expression profile of the nervous and muscular systems (Fig. 1C and Supplementary Table 3). Although almost all the mitochondrial proteins (i.e., proteins that have an expression value from 5 to 0.02 according to the TISSUES database) are expressed in both muscle and nerve cells (Fig. 1D), only 572 mitochondrial proteins have an expression value greater than 2.5 in the muscle tissue. In contrast, 1054 of the 1275 nodes are highly expressed in the nervous system (Fig. 1E and Supplementary Table 3), further confirming the impact of mitochondrial functions on the maintenance of neuronal homeostasis.

In line with the different mitochondrial proteomics profiles [20] and the ability to synchronize their activity in human tissues [21, 22], mitochondrial communication goes beyond the simple cellular boundaries and reaches an inter-organ range. Indeed, under stress conditions, mitochondria release specific signaling molecules, called mitokines. These molecules are able to communicate in a paracrine manner the local mitochondrial stress, inducing an analogous stress response or a metabolic adaptation in receiver cells [10]. As an example, the mitochondrial-derived peptides MOTS-c, Humanin, and Adrenomedullin 2 (ADM2) have been classified as mitokines since they are able to impact whole-body physiology by targeting specific cell types or organs [29–32]. The recent discovery of an altORF in the human mitochondrial *nd4* gene—which generates a micro-peptide (i.e., MTALTND4) able to impact cell and mitochondrial physiology—opened new horizons for the discovery of other mitokines with a paracrine effect [33]. Given the importance of these secreted mitochondrial peptides, it is not surprising that their alteration has been already linked to many human diseases and to the systemic side effects of mitochondrial dysfunctions [10, 34]. Still, many aspects of mitokine functions have yet to be elucidated, including: (**i**) how different perturbations affect the secretion of a specific mitokine in different tissues, (**ii**) how the activation of these extracellular signals is dependent on the mitochondrial proteomics profile, and (**iii**) how mitokines could be used as biomarkers to profile human physical conditions in health and disease.

### Mitochondrial communication impairments in neurodegenerative diseases

In recent years, mitochondrial alterations have been under investigation for their impact on the functions of other cellular components, as well as for their ability to elicit pathological responses in distant cells or tissues. In the neurodegenerative disease field, this wave of interest has started with the discovery of the immunomodulatory role of secreted mitochondrial damage-associated molecular patterns (mtDAMPs) [35, 36]. Indeed, numerous evidences are now drawing a connection between the increased mitochondrial damage typical of many neurodegenerative disorders, and the pathological activation of the innate immune response. This crosstalk appears to mostly rely on the impairment of mitochondrial function and disposal (i.e., mitophagy), which in turn causes the secretion of mtDAMPs [35, 37]. As an example, the cytoplasmic and mitochondrial accumulation of TAR DNA-binding protein 43 (TDP-43, whose mutations are associated with amyotrophic lateral sclerosis (ALS) and frontotemporal dementia (FTD)) determines the release of mtDNA (one of the best-characterized mtDAMPs [36]) in the cytoplasm. This pathological process activates the cGAS/STING pathway, thus inducing the initiation of the neuroinflammatory response [38]. Mitophagy impairment has also been associated with increased mtDAMPs release and inflammasome activation [35, 37]. Given that altered mitochondrial disposal has been reported in most genetic forms of ALS [39–41], a deeper investigation of mtDAMPs generation, as well as their role in ALS onset and progression is still required.

Besides mitophagy, the generation of mitochondrial-derived vesicles (MDVs) is another important mitochondrial quality control pathway that allows the lysosomal degradation of mitochondrial-damaged cargos in a PINK1/Parkin-dependent pathway [42]. Alterations of this physiological mechanism have been already associated with many neurological disorders [43]. In particular, the loss-of-function of mitophagic proteins PINK1 and Parkin (whose mutations have been implicated in the onset of familial Parkinson’s disease) promotes the release of mitochondrial-derived vesicles (MDVs) and their fusion with late endosomes [44]. This pathological process culminates with the presentation of mitochondrial antigens on the cellular surface (i.e., mitochondrial antigen presentation, MitAP) and the consequent activation of the immune response [44]. As this process is triggered by the alteration of the mitophagic pathway and by the subsequent accumulation of damaged mitochondria [44], its activation should be evaluated in ALS patients with optineurin (*OPTN*) mutations. Indeed, optineurin coordinates the autophagosome engulfment of mitochondria, and its mutation causes a decline in the cellular autophagic capacity [41].

The pathological buildup of dysfunctional mitochondria can also be aggravated by the reduction of lysosomal mass and endoplasmic reticulum (ER)-mitochondria contact sites. These cellular impairments can be caused by the acute mitochondrial stress typically found in many other neurodegenerative disorders [45, 46].

In spite of the pathological mechanisms affecting mitochondrial inter/intra-cellular communications, a common problem in the neurodegeneration research field is the understanding of the primary cause of disease onset, as well as the identification of specific biomarkers for the timely and accurate diagnosis of each neurodegenerative pathology [47]. Thus, the characterization of the organelle alteration timeline in cellular models will become important for the identification of novel disease biomarkers and for the development of effective therapies. The use of patient-derived induced pluripotent stem cells (iPSCs) will facilitate this characterization by allowing the investigation of organelle dysfunctions and communication alterations during cell differentiation [48]. Independently from the type of cellular models used to decipher the timeline of inter-organelle network alteration, the use of high-throughput approaches is now becoming essential for its global investigation.

### New horizons of the mitochondrial communication era

The need to increase our understanding of mitochondria physiological connections and their impairment in disease states has expanded the boundaries of high-throughput methods.

**Fig. 2:**
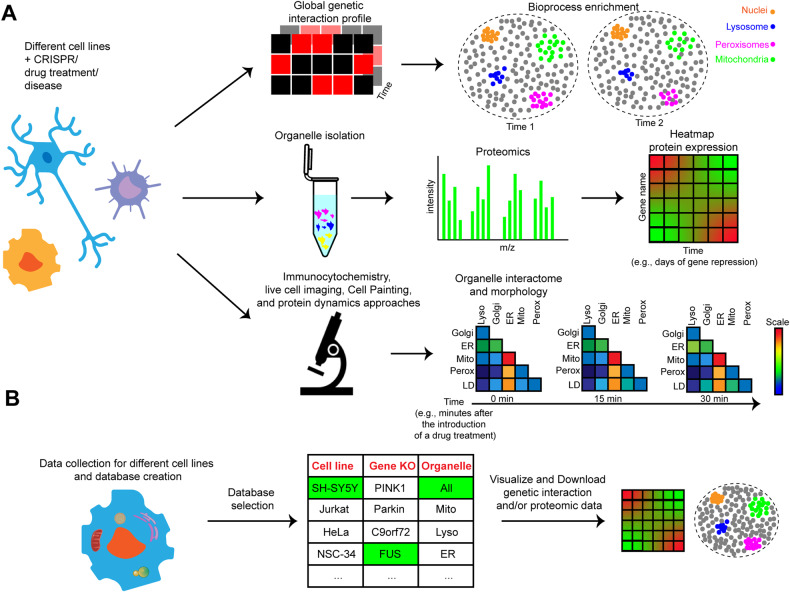
Global approaches to decipher inter-organelle communication. **A** Application and integration of high-throughput approaches for the spatio-temporal characterization of the cytoplasmic organelle crosstalk in different cell lines. **B** Data can be collected from the steps indicated above (**A**) to create a database that will allow the comparison of the genetic interactions and proteomics profiles among different cell lines.

In this context, CRISPR/Cas9-based genome editing approaches have opened the possibility of investigating the phenotypic impact of genetic interactions on a genome-wide scale in model systems. Global genetic interaction screening through CRISPR knockout or interference [49] allows us to quantitatively characterize the functional similarity of gene pairs and to verify how the deletion of a gene pertaining to a specific cellular compartment impacts other organelles’ functionality. This deeper mechanistic insight has already been successfully applied to the budding yeast *Saccharomyces cerevisiae* [50]—it can be visualized as a web-accessible database (https://thecellmap.org/?q=null) [51]—and to human cancer cells [49, 52–54], but it still needs to be implemented on a global scale in other human cellular models (e.g., iPSC-derived neurons). The application of CRISPR interference (CRISPRi) on human-derived cells could indeed reveal clusters of connections between genes pertaining to the same organelle, pathway, or bioprocess, as well as interactions among different cellular compartments (Fig. 2A). This characterization could be further strengthened by the use of different proteomics techniques, allowing multi-omics profiling of inter-organelle communication in physio-pathological conditions. Moreover, after the selection of a gene interaction network or bioprocess of interest, the impact of a specific inducible gene deletion (i.e., inducible CRISPRi [55]) can be evaluated through spatial proteomics at the subcellular level. This approach can depict the remodeling of protein cellular expression in a spatio-temporal manner, for instance, by measuring the protein levels in each cellular compartment after inducing the gene deletion for one or more days [56, 57] (Fig. 2A). The power of spatial proteomics approaches can be exemplified by the hyperLOPIT pipeline, which elegantly couples the TMT labeling of proteolytically digested proteins with the resolution of subcellular compartments’ fractionation, thus allowing the precise identification and quantification of the subcellular proteome [58].

Following the advent of single-cell proteomics, efforts have recently been made to reach an even higher resolution, moving from a single cell to a single organelle level. This innovative approach was first applied for the proteomics characterization of single vesicles secreted from neuronal cells [59], and then adapted for the analysis of single lysosome metabolic profiles [60]. It could however be expanded to the proteomics characterization of other cytoplasmic organelles following the induction of a cellular stress signal or the injection of a specific drug treatment. Ranging from smaller to bigger, spatial proteomics has now been successfully employed to unravel the cellular heterogeneity in three-dimensional (3D) intact samples using a technique called 3D imaging of solvent-cleared organs profiled by mass spectrometry (DISCO-MS) [61].

The recent technological development in cell imaging allows the study of inter-organelle communication at different levels through in-depth image analysis, ranging from the detection of alterations in organelles' morphology to the extensive description of cytoplasmic organelle organization and dynamics (Fig. 2A). For example, the combined application of multiplexed fluorescent dyes and bioinformatic tools allows the morphological analysis of different cytoplasmic compartments and organelles [62]. This phenotypical investigation of organelle morphology is particularly useful in large CRISPR/Cas9 and drug library screenings, where the identification of a pathological phenotypic signature or its rescue represents the main purpose of the study. The effect of gene deletions or drug treatments can also be evaluated at the level of organelles’ contact sites using live-cell microscopy coupled with light sheet spectral imaging, an approach able to give a spatio-temporal resolution of the contact sites [63]. The extent of inter-organelle contact sites can be measured in more targeted ways using the SPLICS (i.e., split-GFP-based contact site sensor) technique [64, 65] or the fluorescent in situ hybridization (FISH) assay coupled with a dual reporter system [66].

In a more global perspective, subcellular connectomics has transformed our perception of cellular structures connectivity not only in the nervous system [67], but also in striated muscle cells [68]. Bleck and colleagues have indeed achieved a deeper understanding of the mitochondrial network connectivity in cardiac, oxidative, and glycolytic muscle by linking mitochondrial network size and shape with the different energy demands of each muscle type, thus providing a map of these organelles’ interactions and distribution across different muscle fibers [68].

Even though different low and high-throughput methods have been used to investigate organelle’s connectivity in health and disease, it is now imperative to implement databases collecting their morphological characteristics, their multi-omics profile in different cell/tissue types, and their alterations in different pathological conditions. This could be achieved by generating CRISPRi genetic interaction network maps of different human cell lines and by revealing the (dis)similarity of cellular compartment bioprocesses in different cellular environments. This characterization can also be matched by in-depth profiling of the physio-pathological proteome of the different cytoplasmic compartments through spatial or single-cell proteomics. Data integration and visualization on a web-accessible database will then allow us to easily evaluate the genetic and proteomic mitochondrial social network in distinctive cell types, as well as disease phenotypes (Fig. 2B).

Altogether, we believe that the use/integration of these global approaches, as well as the creation of web-accessible databases will not only unlock new knowledge on the intra-cellular and inter-organ social interconnectivity but will also uncover new potential therapeutic targets. Lastly, the development of more in-depth approaches will allow the investigation of a possible autonomous regulation of individual mitochondria [54] and their independent crosstalk with other cytoplasmic organelles.

### Supplementary information

Supplementary Information - Bioinformatic analysis

Supplementary Table 1

Supplementary Table 2

Supplementary Table 3
