# Supplementary material for: Keep in touch: a perspective on the mitochondrial social network and its implication in health and disease
Source: Cell Death Discov. 2023 Nov 16;9:417. doi: 10.1038/s41420-023-01710-9 (PMC10654391; doi:10.1038/s41420-023-01710-9)
Supplement: Supplementary file 1 — Supplementary Information - Bioinformatic analysis [file 41420_2023_1710_MOESM1_ESM.docx]

**Keep in touch: a perspective on the mitochondrial social network and its implication in health and disease**

Silvia Barabino^1*^, Silvia Lombardi^1^, Mara Zilocchi^1*^

^1^Department of Biotechnology and Biosciences, University of Milano-Bicocca, 20126 Milan, Italy

^*^ Corresponding author emails: [silvia.barabino@unimib.it](mailto:silvia.barabino@unimib.it) and [mara.zilocchi@unimib.it](mailto:mara.zilocchi@unimib.it)

**Running title:** Mitochondrial crosstalk in health and disease

**Supplementary Information - Bioinformatic analysis**

**UniProt database**

In order to download the UniProt protein IDs pertaining to the eight cytoplasmic organelles (i.e., cytoplasm, endoplasmic reticulum, endosome, Golgi, lipid droplet, lysosome, mitochondrion, and nucleus) chosen for this perspective article, we changed the search bar on the UniProt website (<https://www.uniprot.org>) from UniProtKB to subcellular locations. We then typed the name of the cytoplasmic organelle of interest (e.g., mitochondrion) and chose the specific ID compartment:

- Cytoplasm: SL-0086;
- Endoplasmic reticulum: SL-0095;
- Endosome: SL-0101;
- Golgi apparatus: SL-0132;
- Lipid droplet: SL-0154;
- Lysosome: SL-0158;
- Mitochondrion: SL-0173;
- Nucleus: SL-0191.

After selecting the cellular component and clicking on the “Browse all” icon, the protein list was filtered for status (Reviewed Swiss-Prot only) and organism (human only).

The list of protein IDs specific for each compartment was then download from the UniProt website. We specify that our list of proteins was downloaded on March 24^th^ 2023. Since the UniProt website is updated every eight weeks (<https://www.uniprot.org/help/downloads#:~:text=UniProt%20is%20updated%20every%20eight,using%20ftp.uniprot.org> ), as well as the number of proteins for each specific cellular compartment, the number of proteins might now slightly differ from the one we downloaded to conduct the bioinformatic analysis.

The downloaded protein lists were then used to verify the dual or multiple cellular localization for each UniProt ID (i.e., human protein).

**STRING Cytoscape app**

To verify the mitochondrial protein expression values in 20 different human tissues, we imported the list of all the mitochondrial proteins (Supplementary Table 1) into the STRING app (<https://apps.cytoscape.org/apps/stringapp>) available for Cytoscape (<https://cytoscape.org>) (version 3.9.1).

In particular the mitochondrial UniProt IDs were inserted in the “protein query” function of the STRING app, thus automatically generating a network of interacting proteins. After performing the STRING functional enrichment, we filtered the STRING enrichment table to visualize the data only for the TISSUES database, thus automatically generating a table with the expression values for each mitochondrial protein for all the 20 human tissues listed in Supplementary Table 3. We then visualized the heatmap (Figure 1 C) using the JTree HeatMap View function of the ClusterMaker2 app (https://apps.cytoscape.org/apps/clustermaker2) available on Cytoscape. Lastly, the Venn diagrams (Figure 1 D and 1 E) were created using the website <https://bioinformatics.psb.ugent.be/webtools/Venn/> .
